# Stereoscopic Depth Perception Using a Model Based on the Primary Visual Cortex

**DOI:** 10.1371/journal.pone.0080745

**Published:** 2013-12-05

**Authors:** Fernanda da C. e C. Faria, Jorge Batista, Helder Araújo

**Affiliations:** Institute of Systems and Robotics, University of Coimbra, Coimbra, Portugal; McGill University, Canada

## Abstract

This work describes an approach inspired by the primary visual cortex using the stimulus response of the receptive field profiles of binocular cells for disparity computation. Using the energy model based on the mechanism of log-Gabor filters for disparity encodings, we propose a suitable model to consistently represent the complex cells by computing the wide bandwidths of the cortical cells. This way, the model ensures the general neurophysiological findings in the visual cortex (V1), emphasizing the physical disparities and providing a simple selection method for the complex cell response. The results suggest that our proposed approach can achieve better results than a hybrid model with phase-shift and position-shift using position disparity alone.

## Introduction

Physiologically inspired models of binocular disparity estimation tend to consider the information represented by neurons in primary visual cortex (V1) as an elaborated form of an energy model. Previous studies to extract stereoscopic depth from retinal disparity established Gabor functions as the standard computational model of V1 cells. They represent the finely tuned depth perception cells, i.e., complex cells, by two pairs of 2D Gabor filters with a quarter-cycle shift between phases, calculated by sine and cosine Gabor functions.

Understanding how the complex cells are described by Gabor filters and considering issues such as DC component for larger bandwidths, the phase imbalance for quadrature relationship by sine and cosine waveforms, and the asymmetric frequency response on a log axis, we propose a mathematical representation of complex cells to improve the errors introduced by these three features mentioned, using logarithmic Gabor functions with Hilbert transform. Our analysis and computer simulations show clear evidence of contributions of log-Gabor and Hilbert transform as an appropriate direction for V1 cells representation.

Using hybrid models with phase-shift and position-shift has become a common standard computational model to estimate disparity maps. We explore binocular images in natural viewing conditions of position-shift and our method predicts better disparity maps than hybrid models, without combinations of inter-ocular phase-shift (unnatural disparities). In our case, considering the response cell with the highest local extremum allowed us to identify the correct disparity.

Our work is structured as follows: first, in the Visual Biological Model section, a neurophysiological description of stereoscopic vision theory consistent with V1 cells information is given. Second, in the section dealing with Modeling Stereo Disparity Estimation, a mathematical analysis of complex cells, represented by log-Gabor functions and a comparison with Gabor functions features, is performed. We focused on the computation of position-shift for stereoscopic depth perception. Third, in the Results section, we compute the disparity map for examples of synthetic stereograms with small and large disparities and real world stereograms. Finally, we discuss the advantages and precision of our proposed method and we conclude by presenting the results obtained.

## Visual Biological Model

Depth perception in the brain occurs as a result of the horizontal separation between the eyes. The different locations on the two retinas are crucial to detect variations in depth within the scene. Binocular disparities are the positional displacements between corresponding features in the pair of stereo images. The brain uses the two-dimensional retinal images to understand stereoscopic depth. The three-dimensional properties of the world are coded in the primary visual cortex (V1), based on the known properties of their cells [1]–[3].

Computational theories of vision involving relevant neural mechanisms can simulate implementations from the V1 to find binocular fusion and stereopsis [3]–[5]. An important issue for understanding the physiological approaches is to consider binocular cells to encode disparity, different from nonphysiological algorithms based on matching properties from each monocular left- and right-eye images. Those numerous techniques estimate the correct set of corresponding points under mathematical formulations that cannot be performed by the brain [6].

To interpret visual information, bio-inspired models use the response of the 2D receptive field (RF) profiles of binocular cells to estimate the disparities. Some neurons found in V1 are linear and many nonlinear, with tuning properties for several attributes. There are two types of cortical cells involved in the estimation of the disparities: simple cells and complex cells. Simple and complex cells are made up of orientation selective RFs. Simple cells have antagonistic and spatially separated regions. These regions have distinct responses to a stimulus (excitatory or inhibitory). On the contrary, complex cells do not have spatially separate regions and they respond to a stimulus anywhere within the RF. These characteristics make complex cells finely tuned to binocular disparity [3], [7]–[10].

**Figure 1:**
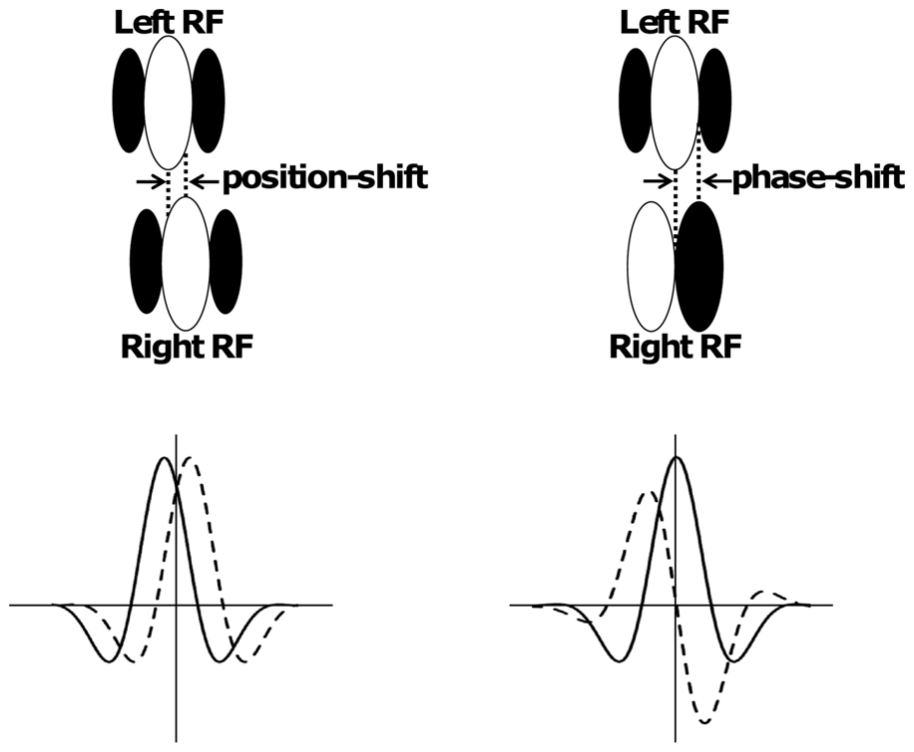
Disparity tuning curves of a pair of receptive fields (Left RF solid lines; Right RF dashed lines) of binocular simple cells with position shift (left) and phase shift (right). Position-shift disparity tuning curves have identical shapes and a horizontal translation between them. Phase-shift disparity tuning curves have different shapes and the same location in the two eyes.

The mechanisms to encode useful information about disparities aim to examine simple cells RF with shifted phase and position. RFs are shifted in position when the left and right RFs of a simple cell have the same shape, but centered at different spatial locations. RFs are shifted in phase when the left and right RFs of a simple cell have different shapes but are in the same spatial location [1], [3], [11]. Figure 1 (adapted from [1], [4]) shows both position and phase disparity mechanisms. In the initial stages of the brain these two types of stimuli studied (i.e., position-shift and phase-shift) are physical disparities and nonphysical disparities. One occurs in the real world naturally and it is characterized by pure position disparities. The other is simulated in laboratory artificially and it is characterized by pure phase disparities [12].

Phase disparities of the tuning curves were classified according to their responses on the two retinas. Binocular interaction with zero phase disparity in both eyes is classified as tuned excitatory (TE) neurons and the disparity tuning function shows an even symmetrical response profile. Cells with RF phase disparity of 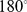 are classified as tuned inhibitory (TI), and like TE-type, the response profile is even symmetric, but inverted. The odd symmetric tuning curves (i.e., asymmetric) have the RF phase disparity of 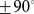, and the depth-sensitive cells are classified as near or far [1], [11], [13].

**Figure 2:**
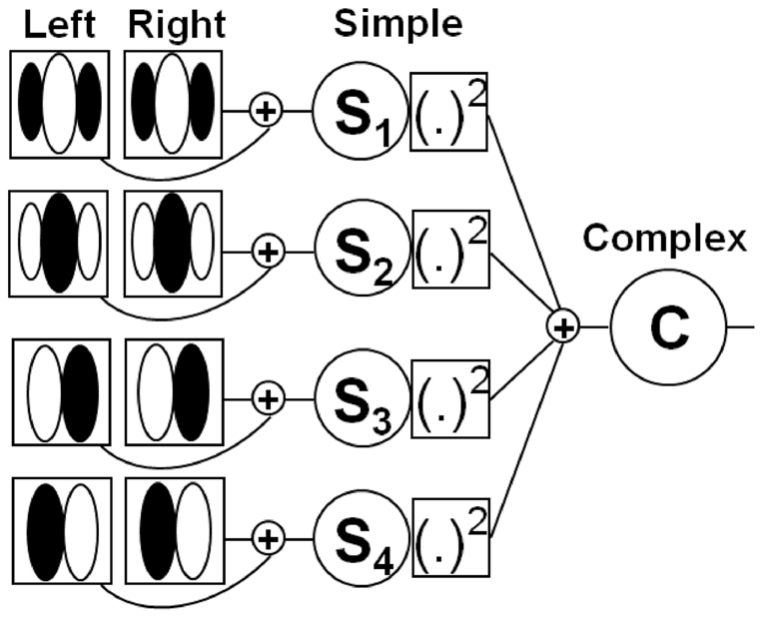
A model of a complex cell. The response of a binocular simple cell is described by the summation of the outputs of their left and right eye filters. The RF configuration is consistent with ON and OFF regions. Complex cells are modeled as nonlinear binocular interactions outputs of two quadrature pairs of simple cells. 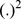 denotes a squaring operation for each matrix element. The final complex cell is arranged as the energy model (sum of squares).

The structure described for complex cells is consistent with an energy model proposed by [14] for motion, and its variant for a stereo matching problem is now being used extensively [3], [15]–[18]. The components of a complex cell are four and they are functionally equivalent to simple cells. The standard model of the simple cell is based on a linear band-pass binocular filter. The response of a binocular simple cell is described by the summation of the outputs of its left and right eye filters. Complex cells are modeled as nonlinear binocular interactions (Figure 2). Their responses can be computed using the squared sum of an even and an odd component, i.e., depth-sensitive cells with RF phase disparity of 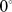 and 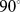 (cells in quadrature) [9], [11], [19].

The example in Figure 2 is a model for disparity selectivity in complex cells adapted from [3], [15]. The model describes the contributions of the left and right RFs as inputs of four binocular simple cells, and their rectified outputs constitute the final complex cell. The RFs profiles illustrated for each binocular simple cell are identical in both eyes. The spatial distribution arrangement of the RFs can vary according to excitatory (ON) and inhibitory (OFF) regions [9]. Excitation increases in the selectivity RF model when inhibition decreases and vice versa. The set of simple cells are arranged into two push-pull pairs (labeled 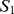 and 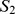; 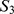 and 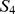), because they cannot fire negative values. Simple cells with low spontaneous firing rate are common. As we show in Figure 2, each simple cell pair configuration has inverted RF profiles, therefore, cells can preserve bipolar signals information. The two pairs of simple cells are also a quarter-cycle shift between ON and OFF regions, i.e., they are in the quadrature phase. The pairs of quadrature simple cells have spatial RF profiles which share a common space location and the same preferred orientation [3], [15], [20].

Several works in the literature compute the binocular disparity in real world images considering the energy model [21]–[24]. They provide a representation of the visual cortical cell modeled by Gabor functions [10] to solve the stereo correspondence problem. Although these descriptions have been widely adopted, this paper aims to examine log-Gabor functions to achieve a mathematical description of the initial stages of the brain's stereo algorithm.

In [21] an algorithm is described that uses a coarse-to-fine procedure with both phase-shift and position-shift receptive fields mechanisms for processing of binocular disparity. The response of a binocular simple cell is computed by using a 2D Gabor functions. In spatial pooling a 2D Gaussian function is used to combine the responses of quadrature pairs around each location. Orientation pooling is performed by means of the average of the population response curves within the full range of orientations. The peak location of the averaged curve is computed by a parabolic fit of three points around the peak. The results suggest that phase-shift RF mechanism is better suited for disparity computation than the position-shift mechanism.

The work in [22] estimates the disparity using a population of hybrid position-disparity and phase-disparity neurons. The Gabor RFs are shifted symmetrically in opposite directions. The stimulus disparity can be found at a local extremum (maximum or minimum) of the population response. The characteristics of the extremum points are as follows: the position disparities should be at a local maximum or a local minimum, and the phase disparities should be at a local maximum. The maximum and minimum points selected are used to compute the extremum point using the Nelder-Mead algorithm. The resulting maps of different spatial frequencies and orientation channels are pooled using a simple robust-averaging heuristic. Using this method, and for uniform-disparity stimuli, the algorithm is guaranteed to find the correct disparity, even within a single spatial-frequency and orientation channel.

The authors in [23] adopt an algorithm for disparity estimation based on a confidence measure that uses a population of hybrid disparity neurons tuned to different phase-shifts and position-shifts. The confidence measure is used to classify the stimulus disparities as inside or outside the range of preferred disparities in the population. The classification error is reduced applying Bayesian classifiers. The position-shift selection involves a winner-takes-all network and the estimated disparity is improved using spatial and orientation pooling. The paper suggests that the pixels with low confidence are likely to be in occluded regions.

The algorithm presented in our previous work [24] is a bio-inspired computational model to compute binocular disparity with position-shift receptive field. The binocular energy model employed two Gabor functions whose phase value is always zero. The initial values used by the algorithm are based on the most responsive complex cells. After the first spatial frequency, the complex cells responses at a local maximum or a local minimum are selected. The resulting maps with different spatial frequencies and RF orientations are pooled.

This work is an alternative approach to investigate the mechanism of disparity encoding of the log-Gabor filters [25]–[27], that support general neurophysiological findings in V1 neurons. We developed a model to consistently represent the complex cells with a suitable filter (log-Gabor) to compute the wide bandwidths of the cortical-cells. The model proposed emphasizes the physical disparities unlike the others models [21]–[23] that depend of nonphysical disparities to decode images. Our model facilitates computation and implementation of the disparities by providing a simple selection of the complex cells responses. The results achieved are better for the same three images from Middlebury stereo repository [6] than the results showed in [22] whose algorithm is very time-consuming. We also provide results computing disparity maps for random dot stereograms with the same characteristics shown in [21] and our algorithm presents good results. Finally, there is another important factor that needs to be considered, which is that our proposed algorithm always shows better results with log-Gabor filters than with Gabor filters in equivalent experimental conditions (position-shift disparity, complex cells response selection and estimated disparity maps pooling).

## Modeling Stereo Disparity Estimation

In a mathematical model of depth perception based on primary visual cortex (V1) concepts, the neurons can be treated as 2D filters with a broad range of spatial dimensions, orientation bandwidths and spatial frequency bandwidths [10]. The energy model might be understood as modeling the response of four simple cells by means of two pairs of band-pass filters in quadrature followed by a half-power function. Gabor and log-Gabor filters encode natural images efficiently when the bandwidth is not more than 1 octave. For larger bandwidths (over one octave), the frequency response of the log-Gabor filter permits a more compact representation [25]. Most of the cells in V1 have bandwidths between 1.0 and 1.5 octaves. The average bandwidth of simple cells and complex cells is about 1.4 and 1.5 octaves, respectively [28]. Therefore, log-Gabor filters seem to be adequate when dealing with bio-inspired models due to the wide bandwidth of the simple and complex cells.

Functions of frequency response of many V1 cells are symmetric on a log axis. Log-Gabor function captures the relative symmetry of the tuning curves on a log axis, differently of a Gabor function. With the constant bandwidths, the Gabor filters responses are redundant at low frequencies and over one octave this characteristic becomes more visible. While using log-Gabor functions, the information is maintained uniformly spread across the scales. In addition, the DC component of the log-Gabor is zero for narrow or larger bandwidths. On the contrary, Gabor filters cannot keep a zero DC component for bandwidths greater than 1 octave. Log-Gabor functions allow obtaining wide band spectral information using filters of minimal spatial extent because they have an extended tail at the high frequency end [25].

In agreement with Gabor and log-Gabor functions characteristics concerning bandwidth, log axis symmetry and DC component, there are evidences that the best-fitting for RFs should be log-Gabor filters. Theoretically, log-Gabor filters provide the most complete description of how to represent a RF and they can work properly to represent depth in an energy model algorithm. Both filters have not been compared in a single study to determine which could provide the best performance. In this work such a comparison is performed (section of Results).

Logarithmic Gabor functions, like traditional Gabor functions, can be used to determine the RFs of simple cells. To do this, instead of using a linear frequency scale, as was done in [21]–[24], we now look at the logarithmic frequency scale [25], [29], [30]. Simple cell RFs computation is modeled through a set of log-Gabor filters in the Fourier domain to analyze the signal. A frequency domain computation is provided by taking the two-dimensional Fourier transform of the space-time RF. We start by converting the filter coordinates from Cartesian to polar coordinates. Each point 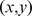 can be described with two polar coordinates 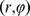. Let 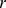 be the radial coordinate (1) and 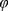 is the anticlockwise angular coordinate (2). In the left RF the positional difference is given by 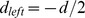 and the right RF is 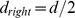, where 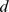 is the positional difference between the left and right RFs.
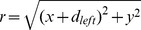
(1)

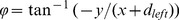
(2)


The two-dimensional filter was built in frequency domain by means of the Fourier transform. It is computed as the product of two separable factors. One term is an angular Gaussian function (3). This function does not affect phase data because functions filtered by a Gaussian undergo amplitude modulation of their components [26].
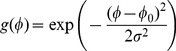
(3)where 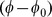 is the absolute angular distance of sine and cosine difference, i.e., 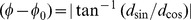 where the coordinate system is rotated according to the orientation angle 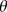 (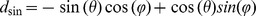 and 
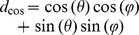
). The 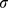 is the standard deviation of the Gaussian function in the angular direction. The standard deviation is constant and its value is 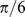 or 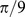, where values are based on the empirical data.

The other term is a variation of the Gabor function where the frequency response is a Gaussian on a log frequency axis so-called log-Gabor filter [25], computed as follows:
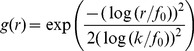
(4)where 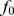 is the central radial frequency, 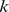 is the standard deviation used to determine the bandwidth of the filter in the radial direction and 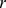 is the radial coordinate (a point in the frequency domain). The term 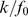 has a fixed value of 0.65 to achieve constant shape ratio filters, i.e., filters that are all geometric scaling of some reference filter. This value will result in a filter bandwidth of approximately 1.5 octaves [26].

The statistics of natural images analyzed in [25] show that scenes from the natural world can be expected to have amplitude spectra falloff of 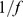. This value is a rough average and in our algorithm we have 4 different spatial frequencies where 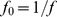.

The overall 2D log-Gabor filter is represented in (5) through the multiplication of the two terms (3) and (4), i.e., the angular Gaussian function multiplied by the log-Gabor filter:
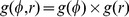
(5)


The response of ON (excitatory regions) and OFF (inhibitory regions) simple cells pair (8) is obtained by the squaring nonlinearity of the half-wave rectified sum of the results of multiplication of the 2D log-Gabor filters with the left (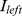) and right (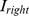) images in the frequency domain ((6) and (7)). The inclusion of OFF simple cells is consistent with Figure 2 as described previously. The basic idea behind combining simple cells pairs of opposite RF profiles is to remove the half-wave rectification to analyze the signal [3], [15], [17]:
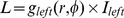
(6)

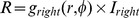
(7)

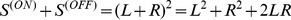
(8)


The binocular interaction RF of a complex cell is given by the sum of the responses in quadrature of two push-pull pairs of binocular simple cells:
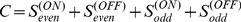
(9)


The two pairs of simple cells in quadrature means that the filters are orthogonal in phase, i.e., filters which have a 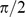 phase shift, while all the other parameters of the cells are identical. Complex cells constructed with Hilbert transform are phase invariant. This is not the case when sine and cosine Gabor functions are used in a quadrature phase relationship, since the two functions have different frequency amplitudes at zero spatial frequency [19], [25], [30].

Equation (9) was rewritten in (10) to detail the application of the filters in quadrature. The responses of an even filter (11) and an odd filter (12) are given by:
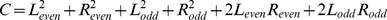
(10)

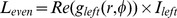
(11)

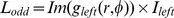
(12)where the imaginary part 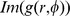 is the Hilbert transform of the real part 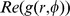.

The fitting algorithm used in our study provides an estimated disparity map created by pooling maps with the same positional differences range (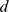) and different spatial frequencies and orientations.

Binocular disparity may be encoded first at low spatial frequencies (coarse scales) and then at high spatial frequencies (fine scales), i.e., a coarse-to-fine algorithm [21], [22]. Disparity is estimated by determining the local extremum of the response of complex cells computed in (9). The extremum is located at a maximum or a minimum response of complex cells for a range of positional differences with the same orientation and spatial frequency [22].

The determination of the local extremum depends on the spatial frequency. If the spatial frequency is the first of a range, then the complex cell selected is the one with the biggest extremum response value. Otherwise a procedure to determine the values of disparity selects the extremum with the closest value to the previous map computed. All values of disparity corresponding to the local extrema selected are saved in a map. The responses of the complex cells are computed for a range of orientations and spatial frequencies. The outputs of the algorithm are a map for each combination of orientation and spatial frequency.

The final disparity map is created by pooling all maps. The method used for pooling is the method described in [22]. Briefly, each spatial position of the disparity map is combined to compute the average. The resulting average is compared with each spatial position of all maps and the position whose value is farthest from the mean is removed. The process is repeated until the total of spatial positions is reduced by half. The final disparity map is more accurate than those that would have been obtained without the pooling step.

The specific parameters used to compute the disparity map for synthetic stereograms and real world stereograms are given in the Results section according to the tests performed.

## Results

**Figure 3:**
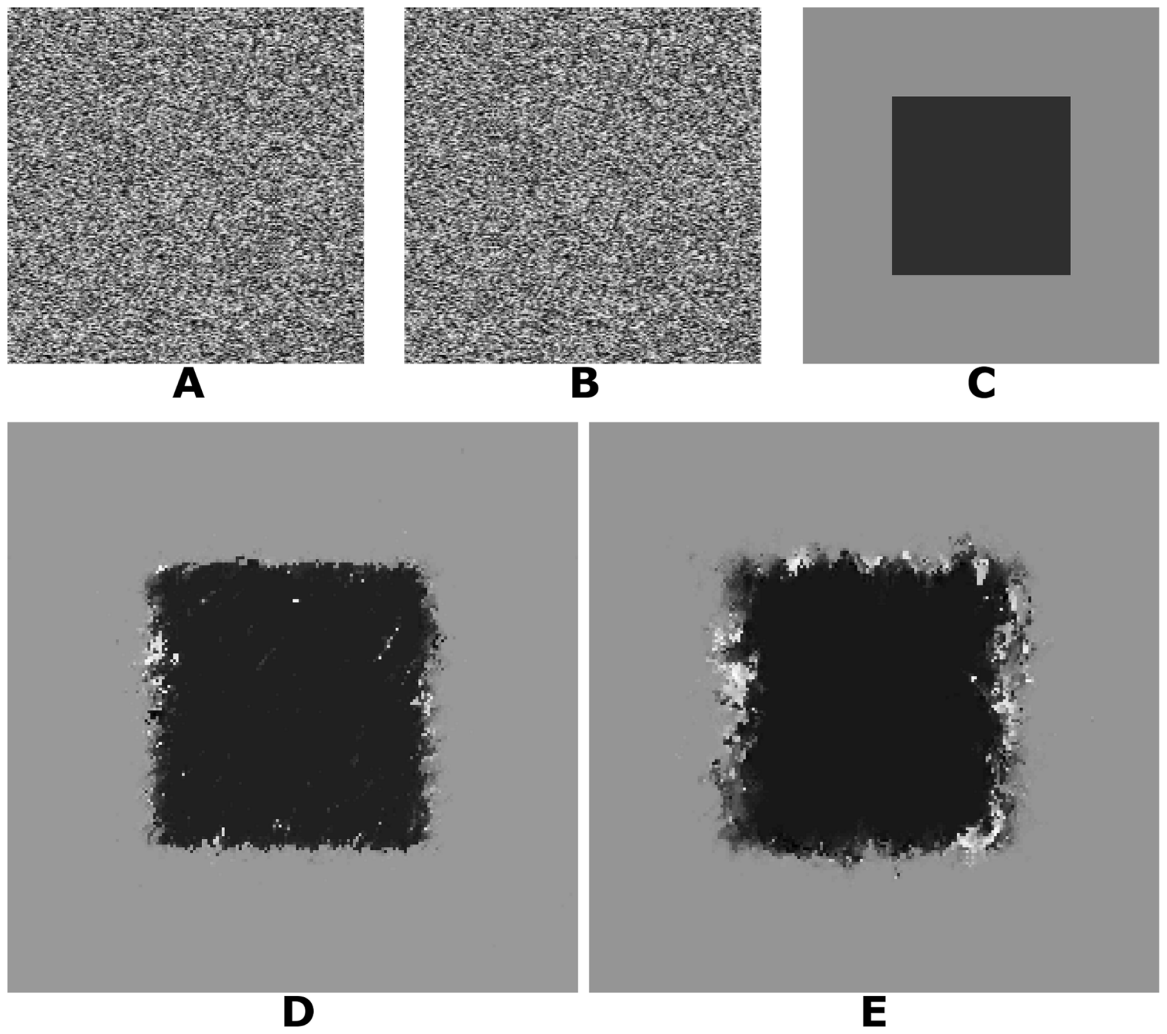
Square stereogram. A: Left image. B: Right image. C: Ground truth disparity map. D: Disparity map using log-Gabor filters. E: Disparity map using Gabor filters.

**Figure 4:**
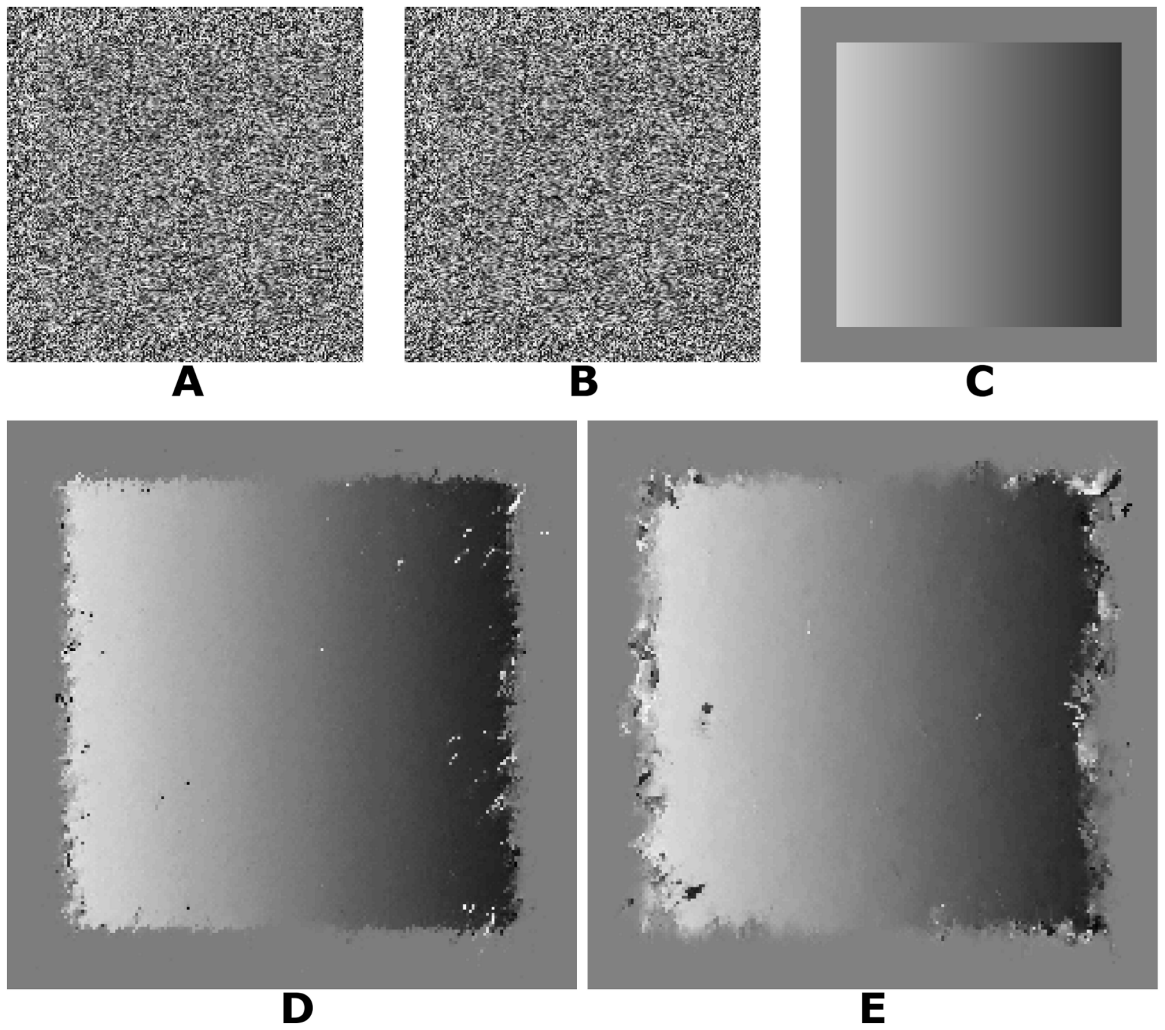
Ramp stereogram. A: Left image. B: Right image. C: Ground truth disparity map. D: Disparity map using log-Gabor filters. E: Disparity map using Gabor filters.

**Figure 5:**
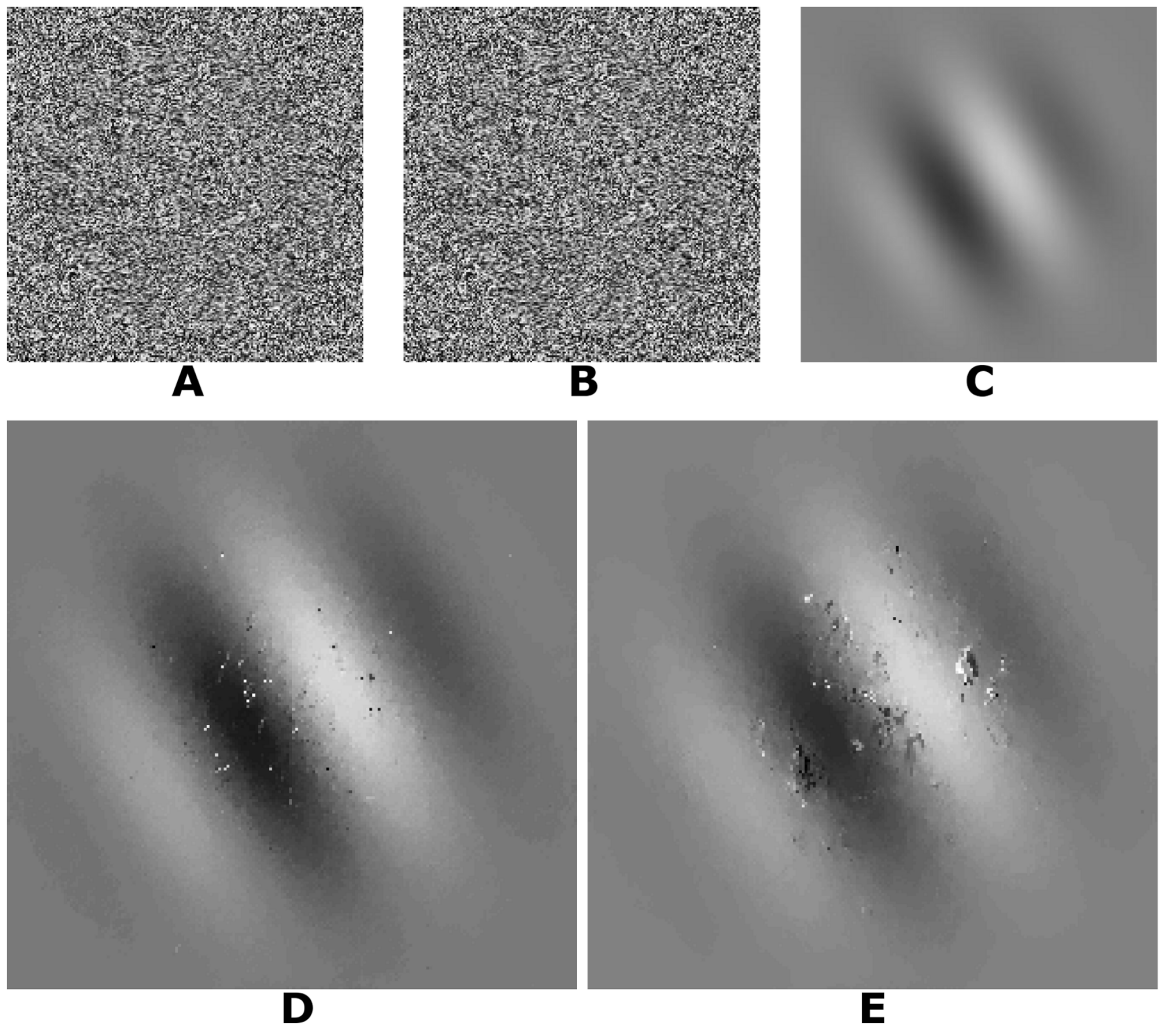
Gabor stereogram. A: Left image. B: Right image. C: Ground truth disparity map. D: Disparity map using log-Gabor filters. E: Disparity map using Gabor filters.

We applied our algorithm to three different kinds of random dot stereograms with a size of 200×200 pixels. The square stereogram uses a disparity of 5 pixels for the central region of 100×100 pixels and a disparity of −1 pixel for the surrounding area (Figure 3). The ramp stereogram is created with disparity varying linearly from −5 to 5 pixels in the central area of 160×160 pixels and a surrounding area with a zero disparity (Figure 4). The Gabor stereogram is constructed using a Gabor function (see Supporting Information File S1) multiplied by a maximum disparity of 5 pixels (Figure 5). The parameters of the Gabor function are: 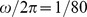 cycles per degree, 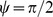, 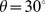 and 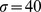 pixels. The tests were performed on a set of 1000 synthetic stereograms of different dot patterns for each kind of stereogram described. The random dot stereograms used are examples of small (1 pixel) and large (5 pixels) disparities.

To compute the disparities for the random dot stereograms we used 6 RF orientations and 4 spatial frequencies. The filter bandwidth is 1.5 octaves. The RFs were computed in a 2D region of 49×97 pixels. The positional differences have a range of 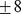 pixels with a step of 1.0 pixel for square stereograms and 0.25 pixel for ramp and Gabor stereograms. For log-Gabor filters the standard deviation is 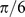, the orientations are 30, 60, 90, 210, 240 and 270 degrees and the spatial frequencies are defined for 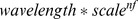, where 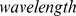 is 3 pixels (wavelength of the smallest scale filter), 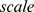 is 1.6 (a scaling factor between successive filters) and 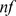 varies from zero to 3 because we have 4 spatial frequencies. For Gabor filters the spatial frequencies are 0.5, 1.0, 2.0 and 4.0 cycles per degree, the orientations are 30, 60, 90, 120, 150 and 180 degrees and the DC components in the stereograms are attenuated by subtraction of the mean luminance from each one before filtering.

Figures 3(D) and 4(D) show that the log-Gabor functions work well on these boundary stereograms when compared to figures 3(E) and 4(E) in which boundaries delivered by Gabor functions are much more blurred.

Table 1 presents the performance of our algorithm employing log-Gabor filters and Gabor filters for synthetic stereograms. The methodologies of evaluation (see Supporting Information File S2) used are percentage of wrongly matched pixels (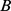) and root-mean-squared error (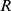). Each table result is a mean of 1000 random dot stereograms. The percentage of wrongly matched pixels is calculated for regions where the disparity estimates exceed 0.25 pixel of error in the disparity.

**Table 1:** Random dot stereograms.

	Log-Gabor Filter		Gabor Filter	
	*B*	*R*	*B*	*R*
Square stereogram	6.18	0.95	10.18	1.14
Ramp stereogram	9.73	0.87	14.29	0.98
Gabor stereogram	7.28	0.25	9.51	0.34

According to Table 1 the proposed algorithm has similar or better results when compared to the estimated disparities in [21], which show a percentage of wrongly matched pixels of 11% and 7% for ramp and Gabor stereograms, respectively. In [21] there are no results for 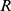 to any stereogram and the square stereogram does not present results for 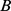.

**Figure 6:**
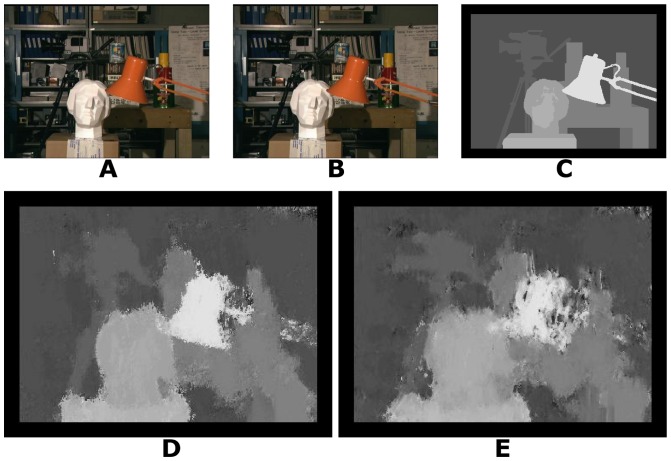
Tsukuba stereogram. A: Left image. B: Right image. C: Ground truth disparity map. D: Disparity map using log-Gabor filters. E: Disparity map using Gabor filters.

**Figure 7:**
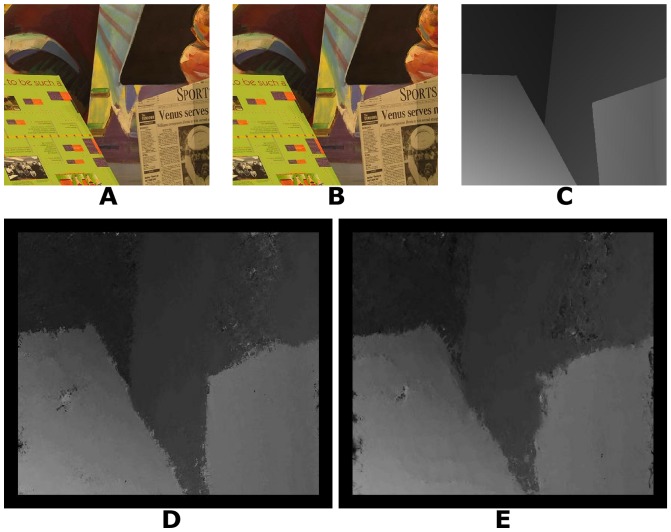
Venus stereogram. A: Left image. B: Right image. C: Ground truth disparity map. D: Disparity map using log-Gabor filters. E: Disparity map using Gabor filters.

**Figure 8:**
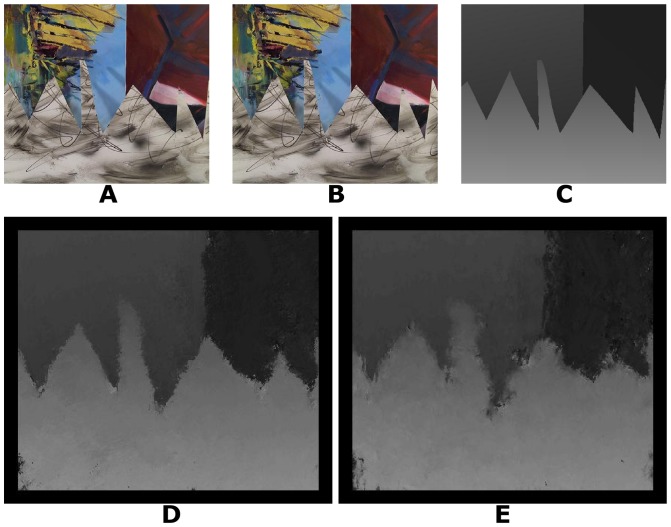
Sawtooth stereogram. A: Left image. B: Right image. C: Ground truth disparity map. D: Disparity map using log-Gabor filters. E: Disparity map using Gabor filters.

We tested our algorithm with 25 real world stereo-pairs (three examples are shown in Figures 6, 7 and 8) from the Middlebury stereo repository (http://vision.middlebury.edu/stereo/) [6], [31], [32].

To compute the disparity images for the real world stereograms, the values used for the parameters were, for almost all parameters, similar to those used for the random dot stereograms. The positional differences have a step of 0.5 pixel and range of 0 to −15 pixels for the image presented in Figure 6 and a range of 0 to −19 pixels for the images presented in Figures 7, 8 and all other images used for the results shown in Table 2, according to the ground truth. The results shown in Table 2 with images from [31], [32] are stereograms files formed by views 1 and 2. The standard deviation used for the log-Gabor filters is 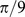 and the spatial frequencies are defined with a scaling factor of 2.1. The parameter values were chosen empirically, based on the best results obtained.

**Table 2:** Real world stereograms.

	Log-Gabor Filter		Gabor Filter	
	*B*	*R*	*B*	*R*
Tsukuba stereogram	15.79	1.60	20.72	1.71
Venus stereogram	10.83	1.24	14.03	1.37
Sawtooth stereogram	9.72	1.76	14.40	1.94
Aloe stereogram	14.56	1.33	19.10	1.37
Baby1 stereogram	6.83	0.93	10.37	1.03
Baby2 stereogram	8.26	0.89	11.44	0.99
Baby3 stereogram	11.14	0.93	14.75	1.10
Barn1 stereogram	10.02	1.54	15.51	1.77
Books stereogram	8.68	0.95	12.14	1.26
Bowling1 stereogram	19.04	2.15	25.97	2.36
Bowling2 stereogram	15.01	1.46	21.28	1.76
Bull stereogram	6.80	0.79	8.41	0.84
Cloth1 stereogram	0.76	0.26	0.86	0.26
Cloth2 stereogram	7.56	0.91	9.73	1.27
Cloth3 stereogram	3.73	0.54	5.31	0.60
Cloth4 stereogram	6.82	1.31	9.94	1.54
Dolls stereogram	11.23	0.72	14.32	0.92
Flowerpots stereogram	12.25	1.06	19.04	1.33
Laundry stereogram	16.31	1.36	20.34	1.49
Moebius stereogram	14.28	0.89	18.19	1.06
Reindeer stereogram	20.45	1.94	26.12	2.05
Rocks1 stereogram	4.85	0.48	6.20	0.64
Rocks2 stereogram	2.30	0.38	3.42	0.43
Wood1 stereogram	9.41	1.86	12.19	2.07
Wood2 stereogram	14.04	1.88	20.70	2.14

Table 2 shows the performance of our algorithm with real world stereograms. The methodologies of evaluation are the same as Table 1. The percentage of wrongly matched pixels is calculated for regions where the disparity estimates exceed 1 pixel of error in the disparity. We also excluded border pixels because the algorithm does not compute meaningful disparities near the image boundaries (border regions of 18 pixels).

According to Table 2, the proposed algorithm has better results than the estimated disparities in [22] which shows a percentage of wrongly matched pixels of 30%, 13% and 21% for Figure 6, Figure 7 and Figure 8, respectively, and a root-mean-squared error of 2, 1 and 2 for Figure 6, Figure 7 and Figure 8, respectively.

Log-Gabor filters always show better results (Table 1 and 2) than Gabor filters in equivalent experimental conditions. Another important fact is that our algorithm with Gabor filters has presented better results for Tsukuba and Sawtooth stereograms than the algorithm in [22].

## Discussion

We provide a biologically plausible computational method for computing disparity maps from stereo images that fulfill the requirements of V1 cortical-cells. Our approach uses a complex cell model that is sensitive enough to encode small and large disparity information in synthetic and real world stereograms (using log-Gabor functions).

The results have some interesting implications that may contribute to our work, since the binocular disparity is encoded mainly through RF position disparity, considering neuroscientific relevant features of an energy model. Our biological visual strategy is a simple method for finding optimal local extrema that capture image properties of depth perception. The calculations for local extremum were performed for different spatial locations and the phase-shift is performed for quadrature pairs of simple cells only. We aimed to find a model with good capabilities for the determination of disparities that is not based on a range of phase differences.

The results with logarithmic Gabor function for synthetic and real world stereograms may help explain why properties such as the efficiency with broad bandwidths, no DC component, and the symmetric frequency response on a log axis, are helpful for the extraction of disparity information from stereoscopic images. Another possible factor that may be relevant is the quadrature relationship with Hilbert transform that is phase invariant. These considerations lead us to conclude that the log-Gabor functions are adequate to represent an efficient arrangement of complex cells more accurately than the common representation encoded by Gabor functions.

Improved estimates of the percentage of wrongly matched pixels and root-mean-squared errors are achieved by decreasing the number of spatial frequencies to 4, if we compare to other algorithms [21], [22] that use 5 or 6 spatial frequencies. Our algorithm also converges quickly because of the efficiency and simplicity of computation of the local extremum. An advantage of our method is the reduction in computational processing time and the increasing accuracy of estimated disparity maps.

The signal processing algorithms were implemented in C/C++ with CUDA (Compute Unified Device Architecture). The tests were performed using an Intel quad-core (i7) and a graphics processing unit Geforce GT 230M. The operating system used was Windows 7. Our algorithm spent 5, 16, 16 and 15 seconds to obtain the disparity maps for Figures 3, 4, 5 and 6, respectively. Figures 7 and 8 required more time because of their size and bigger disparity range. We spent 3 minutes and 31 seconds for Figure 7 and 1 minute and 7 seconds for Figure 8. Tests with other algorithms [21], [22] always require more time.

Therefore, our results imply that an algorithm for disparity computation with position-shift alone can provide reliable and accurate estimates. Log-Gabor is an appropriate functional form that contributes to stereoacuity without a hybrid mechanism of phase- and position-shift. In other words, our economic position-shift model with log-Gabor provides a significant improvement in the estimated disparity maps.

## Conclusion

We searched for a satisfactory bio-inspired computational model to provide a consistent perception of depth for the observed behavior of neurons found in V1 in the neuroscientific relevant studies [3], [12], [16], [17]. Gabor filters and hybrid disparity neurons are generally used in computer vision applications for brain modeling [21]–[23]. We have proposed a model for computing disparity maps from stereograms that is based on cortex cells represented by log-Gabor functions.

In this paper, we have shown that RF position disparities are sufficient to encode binocular disparities. In addition our algorithm does not depend on the range of RF phase disparities. The study of binocular disparities concludes that log-Gabor filters facilitate the extraction of true targets to achieve a consistent perception of depth. Our results reflect how well the proposed methodology represents a successful prediction of the disparity information aspects by applying a quantitative evaluation of synthetic and real world stereograms.

In the results presented, we have considered two types of filters (log-Gabor and Gabor filters) to determine a good model of the complex cells response. Tables 1 and 2 show that log-Gabor filters always provide better or similar results when compared to the other hybrid algorithms. Our modifications in the energy model and in the response selection provide better estimated disparity maps.

## Supporting Information

File S1 **Gabor Function.**
(PDF)

File S2 **Equations for the Evaluation of Results.**
(PDF)
