# Supplementary material for: Stereoscopic Depth Perception Using a Model Based on the Primary Visual Cortex
Source: PLoS One. 2013 Dec 5;8(12):e80745. doi: 10.1371/journal.pone.0080745 (PMC3855160; doi:10.1371/journal.pone.0080745)
Supplement: File S1 — Gabor Function. (PDF) [file pone.0080745.s001.pdf]

Appendix S1: Gabor Function

We have applied the same concepts considered in the stereo disparity estimation, but instead of log-Gabor functions, Gabor functions were used to determine the RFs of simple cells:

$$g(x, y, \psi) = \exp\left(-\frac{x'^2 + y'^2}{2\sigma^2}\right)\cos(\omega x' - \psi)$$

where ω is the cell preferred spatial frequency $(2\pi f)$, ψ is its phase, $x' = x\cos(\theta) + y\sin(\theta)$ and $y' = y\cos(\theta) - x\sin(\theta)$ where θ is its preferred orientation. The standard deviation σ can be computed as follows:

$$\sigma = \frac{\sqrt{\ln 2}}{2\pi f} \times \frac{2^\beta + 1}{2^\beta - 1}$$

where the half-maximum bandwidth β is 1.5 octaves.

The response of a complex cell can be modeled by the sum of outputs in quadrature of two push-pull pairs of binocular simple cells. Sine and cosine Gabor functions are used in a quadrature phase relationship.
