# Supplementary material for: Stereoscopic Depth Perception Using a Model Based on the Primary Visual Cortex
Source: PLoS One. 2013 Dec 5;8(12):e80745. doi: 10.1371/journal.pone.0080745 (PMC3855160; doi:10.1371/journal.pone.0080745)
Supplement: File S2 — Equations for the Evaluation of Results. (PDF) [file pone.0080745.s002.pdf]

Appendix S2: Equations for the Evaluation of Results

In order to analyse the results in what concerns horizontal disparities, we have used two measures to compare the disparities map (d_C) with ground truth disparity map (d_T). The percentage of the remaining pixels where the proposed algorithm did not account for the correct disparity is given by B (percentage of wrongly matched pixels) and is computed as follows:

$$B = \frac{1}{N} \sum_{(x,y)} (|d_C(x,y) - d_T(x,y)| > \delta_d)$$

where δ_d is the disparity error and N is the total number of pixels.

The root-mean-squared error is also employed in the algorithm evaluation:

$$R = \sqrt{\frac{1}{N} \sum_{(x,y)} |d_C(x,y) - d_T(x,y)|^2}.$$
